# Within-host dynamics of infection: from ecological insights to evolutionary predictions

**DOI:** 10.1098/rstb.2014.0304

**Published:** 2015-08-19

**Authors:** Olivier Restif, Andrea L. Graham

**Affiliations:** 1 Disease Dynamics Unit, Department of Veterinary Medicine, University of Cambridge, Madingley Road, Cambridge CB3 0ES, UK; 2 Department of Ecology and Evolutionary Biology, Princeton University, Princeton, NJ 08544, USA

The last 20 years have seen a gradual change in emphasis in infectious disease management, driven by two contrasting trends. On the one hand, global initiatives within the UN Millenium Development Goals have had a major impact on the burden of disease around the world [1]. On the other hand, a number of emerging infections have been striking unexpectedly, occasionally reaching pandemic status, but have so far resisted any attempt to predict the nature, timing and magnitude of outbreaks [2]. This has led to a reappraisal of methods for the surveillance, prevention and control of infectious diseases, characterized by contributions from a broader spectrum of disciplines than ever before, with important roles not only for clinicians and public health policymakers but also anthropologists, ecologists and mathematical modellers [3].

The role of ecologists in this endeavour has probably been best acknowledged in the context of zoonotic emergence from wildlife [4], but some ecologists have been engaged in a more profound assessment of the role of parasites^1^ in ecosystems. Indeed, there is growing evidence that parasite diversity is integral to the richness and stability of biological communities [5]. It has even been suggested that the effects of parasite extinctions could be as disruptive as the removal of key predator species [6]. Because examples of the devastating effects of parasite introductions also abound [7,8], the suggestion that parasites could usefully be conserved sounds deeply unorthodox. Yet, ecologists have formulated a credible argument based on an ecological mechanism known as competitive release [9]. It follows from the premise that parasites compete for exploitation of hosts: removing the most successful parasite will allow another one to occupy the vacant niche, potentially becoming an emerging pathogen. For example, concern has been voiced that smallpox eradication has left us vulnerable to the emergence of recombinant orthopoxviruses [10]. Likewise, successful mass vaccination campaigns could facilitate the emergence of ‘underdogs', particularly if the duration or breadth of the antigenic protection conferred by a vaccine is less than that from infection. This could be the case with *Bordetella pertussis* and *B. parapertussis* [11], although there is no clear epidemiological evidence for replacement of the former by the latter [12]. To date, examples of competitive release driving disease emergence are few, arguably owing to the low number of pathogens successfully eliminated. We nonetheless expect that ecological insights into the spread of disease at the population scale will make increasingly important contributions to global health management in the coming years.

Here, we argue for yet another way in which ecological theory, insights and methods can promote the management of infectious diseases: we propose a change of scale and invite the readers of this theme issue on a journey through the ecosystem within. From a parasite's point of view, each host is indeed a highly complex and rich environment where, in addition to nutrients and other vital molecules (e.g. nucleic acids for virus replication), the parasite will encounter varied and at times inhospitable physical conditions, a barrage of immune defences and myriad resident microorganisms. It is therefore not surprising that many ecological concepts have been used successfully to describe and analyse the fate of parasites inside their hosts, generating useful insights for the management of disease. Even in clinical medicine, the impact of the entire within-host community of parasites and commensals on host physiology is increasingly appreciated. For example, the misuse of antibiotics, apart from promoting the evolution of resistant pathogen strains, can have disastrous health consequences by wiping out beneficial members of the microbiota and allowing emergence of pathogenic bacteria in that niche. A striking example is provided by the induction of a ‘super-shedder’ phenotype in mice infected with *Clostridium difficile* following treatment with the clindamycin drug [13]. Another argument for conserving diverse within-host communities focuses upon parasitic helminths, which infect over a third of the human population to this day. The growing success of experimental treatment of auto-immune conditions with live helminths [14] is giving weight to the controversial ‘hygiene hypothesis', which posits that hosts exposed to too few infections become prone to immune-mediated diseases and was first proposed 25 years ago on the basis of a statistical association between eczema and family size (a proxy for exposure to pathogens among children) [15]. It may indeed prove true that some non-zero burden of both germs and worms is optimal for human health. Several contributions to this theme issue illustrate how ecological approaches elucidate the structure and function of such within-host communities.

Understanding the within-host ecology of infection is also an essential step towards predictive models of pathogen evolution. There is presumably no need to reiterate the urgency of the threats posed by the evolution of immune evasion mechanisms and antimicrobial resistance: both topics have been the focus of recent issues of this journal, respectively, Pybus *et al*. [16] and Anderson [17]. While the former explained how the genomics revolution has started to unveil the *patterns* of pathogen evolution [18], providing valuable information about spatio-temporal epidemiological dynamics [19], the latter described the *consequences* of resistance evolution for medical and veterinary management [20], as well as drug development [21]. Complementing those approaches, contributions to the present issue demonstrate how ecological theory can be used effectively to cast light on the evolutionary *processes* at play in infection, chiefly natural selection.

Before outlining the contents of this theme issue, we illustrate the ecological drivers of parasite evolution through a whistle-stop tour of three topics which, albeit crucial to the success of global health management programmes, have been the subject of misconceptions across scientific and medical communities. The resulting controversies have motivated much theoretical and empirical research in the last 30 years. The first one is probably as old as microbiology itself: do pathogens evolve to become less virulent^2^ to their hosts? Microbiology pioneers, including Pasteur [22], were puzzled by the observation that the virulence of microbes isolated from infected animals could increase or decrease within a few passages *in vitro*. Because an immediate consequence of increased virulence is the premature death of the host which, for many pathogens, cuts short opportunities for replication and transmission, an intuitive prediction is that natural selection should favour less virulent strains. Hence, the idea that long-term adaptation to a host should lead to commensal, or even mutualistic, associations. In true Darwinian tradition, Ball [23] was among the first to challenge this dogmatic view, based on careful arguments supported by extensive empirical data. It was not until the 1980s that a solid theoretical framework was developed to generate more detailed and testable predictions for virulence evolution [24,25]. Underlying this breakthrough was the fundamental principle that natural selection does not necessarily favour the strategy that maximizes reproductive success at the population level, but rather the strategy that can beat any competitor within a given ecological context. This means that, if variations in virulence are associated with other traits, such as replication, exploitation of resources, immune evasion, infectiousness or antimicrobial activity, then the optimal balance (as far as natural selection is concerned) will be highly sensitive to the circumstances of competition among parasite genotypes. Indeed, increasingly refined mathematical models have produced a wide range of evolutionary predictions based on subtle variations in their structure or choice of assumptions [26]. To date, the main challenge has been the validation of these models, which requires detailed quantitative information on the genetics of the parasite (good progress has been made there) as well as the within- and between-host dynamics of infection [27]. In conclusion, if the idea that some virulence can be adaptive (i.e. favoured by selection) for most parasites is now generally accepted, the next challenge is to successfully predict and, ultimately, manipulate the evolution of virulence in parasites—one of the seminal goals of Darwinian medicine [28]. Detailed quantitative data on within-host dynamics will be central to achieving that goal.

The second misconception, which has apparently become more prevalent in the medical community, is that pathogens (especially viruses and bacteria) will inevitably evolve resistance mechanisms against any treatment [29]. Most models of antibiotic resistance used to inform public health policies consider resistance as an all-or-nothing property and are calibrated with data from reductionist *in vitro* experiments where large population sizes favour the emergence of mutations conferring resistance [30]. However, the benefits and costs of these mutations measured *in vitro* may not reflect those experienced by the parasites *in vivo* [31,32]. In addition, we are only beginning to assess the contribution of horizontal gene transfer to the spread of resistance within bacterial communities [33]. Assessing the true costs and benefits of antibiotic resistance genes, which are often embedded in larger mobile elements, within the context of the host and its microbiota, is of paramount importance to understand and predict the occurrence of resistance. Even if some form of resistance can appear, there may be ways to prevent or slow down its spread with the help of ecological theory [34]. By combining *in vivo* experiments with ecological modelling, it should be possible to optimize combinations of treatments with a view to make them ‘evolution-proof’. This requires a multi-scale approach that encompasses selection from the level of genes to that of populations. In a different context, phylodynamics provides a clear framework to explain why some viruses (like influenza A or HIV) constantly evolve to evade antigenic recognition, while others (like measles virus) fail to do so [35]. Developing a similar framework for antimicrobial resistance evolution would be a major step forward, and progress will again depend upon rigorous understanding of within-host dynamics.

The third enduring but somewhat misleading view of parasite evolution is that of an escalating arms race with the host, often associated with the Red Queen Hypothesis. Initially proposed in the context of predator–prey coevolution [36], this Cold War-inspired metaphor has been applied to other interspecific associations with variable success. Unsurprisingly, bacteria–bacteriophage systems, which can be seen as hybrids between predatory and parasitic systems, exhibit patterns consistent with arms races [37]. By contrast, parasites that exploit their hosts without killing them can embark on quite different coevolutionary journeys. Although biomedical research on infection tends to focus on the elaborate interactions between immune defences and parasites' counter-measures, these are only part of the parasite's life history. Here too, ecology is key to appreciate the relative importance of different selective pressures. Parasites face a diverse range of threats and challenges, from physical barriers (which can be seen as a front-line extension of the immune system) and strong, specific immunological attack to lack of vital resources and competition with resident microorganisms. In an unexpected twist, to deal with the competition, the enteric pathogen *Salmonella enterica* resorts to triggering an inflammatory response that clears part of the microbiota [38]. In fact, there is growing evidence that various pathogens have evolved to exploit, rather than evade, components of the host's immune response [39]. From the host's perspective too, there are good reasons not to engage in escalating arms races. First, potent immune defences can be more harmful than infection [40] or, as we have just seen, beneficial to pathogens. In addition, because natural selection is essentially concerned with the reproductive success of competing genotypes (and not with the ability to kill pathogens), it may be advantageous for a host to cultivate pathogens that can be used as biological weapons against more susceptible competitors [41]. An iconic example of such a biological weapon is the poxvirus that grey squirrels brought with them from their native America to Great Britain, which has all but wiped out the native red squirrels, facilitating their replacement across the country [42]. On a smaller scale, bacteria have been shown to use lysogenic phages in a similar way [43,44]. Such associations can generate a host phenotype known as ‘tolerance’, by analogy to the strategy used by some plants to cope with herbivores: instead of mounting costly defences to prevent exploitation by these enemies, it may be preferable to tolerate some burden of parasites and invest conserved resources in reproduction [45,46]. In recent years, infection-tolerant phenotypes have been described in a growing number of animal species [47], despite technical challenges. Indeed, in order to quantify tolerance, it is necessary to measure changes in host fitness in response to variations in parasite load, which itself responds dynamically to immune defences [48]. Although theoretical models have been developed to predict the conditions favouring these alternative strategies [49,50], studies of tolerance have yet to take into account the within-host dynamics of infection.

## Outline of the issue

Combining review and original research papers, this theme issue highlights the latest progress in our understanding of the within-host dynamics of infection, including ecological processes and evolutionary consequences of those dynamics. Particular emphasis is given to empirical evidence, in humans as well as animals and plants, in the laboratory or in the field. We have brought together experts from diverse horizons, covering a wide range of host–parasite systems and investigation methods. All the contributions challenge pre-conceived ideas and demonstrate the importance of integrating ecology with other fields for maximal insight into the basic biology and the management of infectious diseases. The Table of Contents lists the contributions in a logical order, as they naturally fall under four topics.

We begin with ecological insights into the interactions between parasites and the adaptive immune system. The first case study investigates the spatial dynamics of cytotoxic T lymphocyte (CTL) response to viral infection: using a modelling framework borrowed from predator–prey ecology, Kadolsky & Yates [51] predicts the effects of spatial distribution of infection and chemotaxis in CTL on the dynamics of virus clearance. Also relevant to T-cell-mediated immunity, the second example [52] addresses the issue of estimating immune repertoire diversity *in vivo* from blood samples. An unexpected solution is provided by ecological methods for species richness estimation. Next, we consider the parasite's point of view, with two contrasted examples of strategies that have evolved to establish persistent infections in the face of adaptive immunity. At one end of the spectrum, HIV thrives in a guerrilla warfare approach, attacking T-cells and constantly evolving [53]. At the other end, trypanosomes manage to keep under the host's immune radar by switching antigens and self-limitation of infection load [54].

Having set the stage of infection dynamics, this theme issue next investigates drivers of parasite genetic diversity and evolution within hosts. Of particular interest is the influence of host factors on competition between parasite strains: following a review highlighting the value of ecological concepts [55], a case study demonstrates how sequencing methods can be used to monitor parasite strain competition in wildlife hosts [56]. The next research paper asks whether mutations that get fixed in the process of virus adaptation to a host genotype all confer fitness advantages in an experimental plant–virus system [57]: surprisingly, some supposedly synonymous mutations appear to have detrimental effects on virus fitness, suggesting an important role of genetic drift at the within-host level. Adding one more layer of complexity, Koskella & Parr [58] tracked coevolution between bacteria and phage within chestnut trees using time-shift experiments and discovered asymmetry in the mode and tempo of reciprocal adaptation between coevolutionary partners.

The next four papers investigate complex dynamics of within-host community ecology, particularly the interaction between the resident microbiota and parasites. Capitalizing on technological progress, Kreisinger *et al*. [59] assessed the relationship between gut bacteriome diversity in wild-caught mice and the presence of various helminths. Their findings suggest complex interactions across the digestive tract. These interactions also appear to play an important role in mediating the effectiveness of helminth-based treatment of auto-immune disorders and chronic inflammatory disease [14]. Albeit less well studied than the gastro-intestinal flora, the respiratory microbiota is beginning to appear as a key player in acute infections: to help tackle these complex interactions, de Steenhuijsen *et al*. [60] proposes an ecological overview of the respiratory commensal bacteria based on the niches they occupy. Finally, McNally & Brown [61] argue that bacterial commensals and parasites do not simply adapt to the diverse environments within their hosts, but they can actively modify them in a process known by ecologists as ‘niche construction’. Using illustrations from three compelling case studies, they highlight the implications of this process for health and disease.

The last part of this issue scales up the ecological framework from within- to between-host dynamics of infection. This scaling-up represents both an enormous challenge and a crucial step for understanding and predicting parasite evolution. In short, from an ecological point of view, a host is an environmental patch in which parasites grow and interact, and it is imperative for the parasite to extract enough from one patch to propagate its descendants to a new patch. For example, one cannot predict the evolved virulence of a parasite strain based solely on its replicative potential within a host; much better predictions arise from additional information on its route(s) and probabilities of transmission to the next host, in relation to how high a within-host density it achieves. To tackle such challenges, contributors begin with a review of the replication strategies used by arthropod-borne viruses in their vertebrate hosts, which can be classified as ‘tortoise’ (low magnitude, long duration viremia) versus ‘hare’(high magnitude, short duration viremia) [62]. Using a mathematical model, the authors propose that ‘tortoise’ strategies may result in more efficient transmission of such viruses. Few experimental models enable tests of such hypotheses, or more generally the investigation of both within- and between-host dynamics and the ensuing parasite evolution. Avian malaria (caused by *Plasmodium relictum*) is one of these. Rivero and co-workers [63] have compiled an extensive dataset on the evolution of *P. relictum* through 5 years of experimental passages in canaries and mosquitoes, providing unprecedented insight into the links between parasite development and transmission. We finish with two theoretical studies that summarize the state-of-the-art of our conceptual understanding of the evolutionary drivers of how parasite transmission arises from within-host dynamics. Handel & Rohani [27] review the empirical evidence and gaps, focusing on the quantitative links between parasite load and transmission. Finally, Alizon and co-workers [64] propose a unified modelling framework for the evolution of transmission in the context of multiple infections.

Each of the contributions to this theme issue represents a major growth area in research and firmly integrates within-host ecology into the global health enterprise. Indeed, within-host dynamics will shape the health of individual hosts, the transmission of parasites across host populations, and our ability to ameliorate either. And ecologists are equipped with the tools and the inclination to characterize such complex dynamics quantitatively. We look forward to the continuation and expansion of this work. The net result will be both broader (e.g. across disease systems) and deeper understanding (e.g. such that we can accurately predict outcomes, and thereby design optimal and evolutionarily sustainable interventions). An integrative interdisciplinary approach with ecology firmly in the fold is, in our view, very much the way forward.

## Endnotes

1 Here, we use the terms parasite and pathogen to denote the same group of organisms with distinct emphases: ‘parasite’ refers to an organism that exploits a host for its own reproduction, whereas ‘pathogen’ refers to the agent of a communicable disease.

2 Here, we use the term ‘virulent’ in the sense of detrimental to the survival or reproduction of the host, in line with standard practice in evolutionary biology. Virulence is thus distinct from (but often correlated to) infectivity and growth rate.
